# Tailoring Super‐Performed Chemo‐Sensor via Simulation‐Modeling and MEMS‐Screening

**DOI:** 10.1002/advs.202412937

**Published:** 2025-01-07

**Authors:** Wei Xu, Wukun Zhang, Zhengqi Shen, Wenxing Xu, Jianhao Zhao, Huizi Li, Qingguo He, Yanyan Fu, Jiangong Cheng

**Affiliations:** ^1^ State Key Lab of Transducer Technology Shanghai Institute of Microsystem and Information Technology Chinese Academy of Sciences Changning Road 865 Shanghai 200050 China; ^2^ Center of Materials Science and Optoelectronics Engineering University of the Chinese Academy of Sciences Yuquan Road 19 Beijing 100039 China

**Keywords:** chemo‐sensor, customized design, micro‐cantilever, structure‐effect analysis, theoretical calculation

## Abstract

Chemo‐sensor designing involves a time‐consuming trial‐and‐error screening process, which commonly cannot lead to optimal S^4^R features (Sensitivity, Selectivity, Speed, Stability, and Reversibility). Due to strong path dependence on reported groups/mechanisms, conventional chemo‐sensors often fail to meet critical application demands, especially in achieving high reversibility without compromising other features. Here, a **three‐step screen and design strategy** is developed for gaining customized chemo‐sensors, through **Structure modeling; MEMS (Micro Electro Mechanical Systems) analysis, and Performance verification**. With such a strategy, the coordination hanging anion mechanism is screened out for reversible nerve agent detection and shows reversible emission enhancement by 25.8 times with DCP, ultrasensitive vapor phase detection (5.7 ppb), and rapid response(10 s) and recovery speed (20 s). Such tailored designing strategy for new organic chemo‐sensors will probably play an important role in developing high‐performance sensing system in the future.

## Graphical abstract

Here developed a three‐step **screen and design strategy for** high‐performance chemo‐sensor, through **Structure modeling; MEMS analysis, and Performance verification**. Coordination hanging anion mechanism is successfully screened out for reversible nerve agent vapor detection, showing 25.8 times reversible emission enhancement, 5.7 ppb ultrasensitive detection limit, and rapid response and recovery.

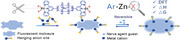

## 1 Introduction

Organic semiconductors can convert trace environmental changes into recognizable optical and electrical signals, which is quite suitable for constructing ultra‐sensitive chemo‐sensors.^[^ ^1^, ^2^, ^3^, ^4^, ^5^, ^6^ ^]^ Compared with conventional techniques from mass spectrometer,^[^ ^7^, ^8^, ^9^ ^]^ biological enzyme method^[^ ^10^, ^11^, ^12^, ^13^, ^14^ ^]^ to ion‐mobility spectroscopy,^[^ ^15^, ^16^, ^17^, ^18^ ^]^ organic chemo‐sensors attracted great attention due to simple operation, real‐time response, non‐destructiveness and device miniaturization. As the most important chemo‐sensor parameters, S^4^R features including Sensitivity, Selectivity, Speed, Stability, and Reversibility, were mainly decided by chemical functional groups.^[^ ^19^, ^20^, ^21^ ^]^ Differences from electronic affinity, and chemical reactivity, to spatial structure, existed among different analysts, even for extremely similar analysts, thus lead to various designing demands for sensitive structures.^[^ ^22^, ^23^, ^24^ ^]^

To obtain chemo‐sensors with specific S^4^R performance, numerous organic functional structures need to be screened for the most adaptive optical/electric performance, and chemical activity. Traditional designing involves a time‐consuming trial‐error screening process full of unexpected factors, and commonly cannot lead to optimal performance.^[^ ^25^, ^26^, ^27^ ^]^ A series of excellent chemo‐sensors have been designed for a wide variety of analytes, from electron‐deficient explosives,^[^ ^28^, ^29^ ^]^ to electron‐rich amine species.^[^ ^30^, ^31^, ^32^, ^33^, ^34^ ^]^ However, simultaneous acquisition of all S^4^R performances, especially reversibility, still remained a challenging task.

For high chemical‐reactive analytes such as organophosphate nerve agents, the strong bonding ability of nucleophilic sites toward phosphorus atoms or protons, results in an irreversible sensing process, while stronger binding interactions lead to a reduction in sensitivity. Organophosphate nerve agents are volatile molecules with phosphate ester structure, which seriously threaten human health and public security.^[^ ^35^, ^36^ ^]^ A series of efficient fluorescent sensors have been gained based on both small molecules and polymers for nerve agent detection, mainly centered on nucleophilic molecular structures.^[^ ^37^, ^38^, ^39^ ^]^ Dominant active mechanism of these structures were strong electric‐negative Nitrogen/Oxygen atoms with lone‐pair electron for constructing host‐guest sites, which can demonstrate a parts‐per‐billion level detection limit.^[^ ^40^, ^41^, ^42^, ^43^ ^]^ Thus, it is in great demand to develop optimized fluorescent structures with both reversible and sensitive nerve agent detection performance.

**Figure 1:**
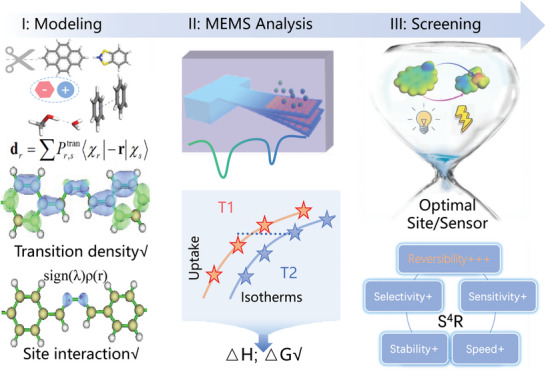
a) Diagram of the three‐step design and screen strategy for organic chem‐sensors, including I.Modelling, II.MEMS Analysis and III.Screening.

Integrated silicon microcantilever resonant device can directly measure thermodynamic/kinetic parameters in adsorption process,^[^ ^44^, ^45^ ^]^ which brings an efficient method for chemo‐sensor screening from potential materials and mechanisms designed through first‐principle calculations. Therefore, here we proposed a rational design strategy for customized chem‐sensors, including 3 steps, as shown in **Figure** **1**.

**I: Modeling**: Create candidate 3D structure models with both common and uncommon groups. Gain transition density and weak interaction distributions of candidate models with theoretical simulation, and choose the promising models.

**II: MEMS analysis**: Extract thermodynamic parameters of adsorption process with MEMS micro‐cantilevers kinetics and further screen out the promising models.

**III: Performance**: Verify S^4^R performance of the screened chemo‐sensor and understand the designed mechanism.

For traditional probes, direct active sites at fluorescent molecule led to irreversible combination with guest molecules. In our mechanism, hanging anions coordinated with fluorescent substrate molecule by metal cations, and acted as active sites with larger contact space for host‐guest interactions.

In addition, the reversibility of host‐guest interactions can be further tuned by designing acid residual anions with variable leaving performance, especially weak acids with reversible ionization ability such as acetic acid.

## 2 Results and Discussion

**Figure 2:**
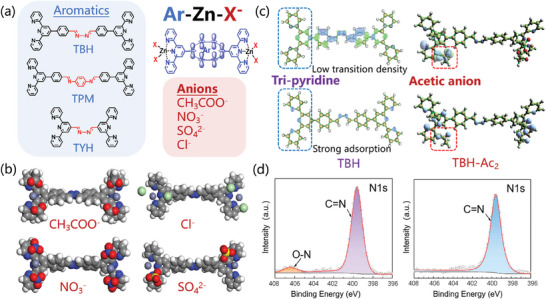
a) The chemical structures of the coordination Aromatic‐Zn‐anion (Ar‐Zn‐X^−^) systems. b) The geometric optimized spatial conformations of different anions coordinated with TBH module. c) Transition density distributions and reduced density gradient (RDG) weak interaction distributions of TBH and TBH‐Zn‐CH_3_COO^−^ (TBH‐Ac_2_). d) XPS spectra of film state TBH‐Ac_2_ before and after interaction with DCP vapor.

In experimental, as shown in **Figure** **2**a, a series of organic coordination models, were established and systematically analyzed with first‐principle calculation to gain targeting S^4^R super‐performance. Tri‐pyridine derivatives were chosen as the substrate molecules to gain various DCP sensing films (TBH, TPM, and TYH with different Schiff‐base connector structures), due to the good photo‐stability and sensitivity according to previous work.^[^ ^37^, ^38^ ^]^ For another major component of the coordination probe model, variable anion species were considered for reversible sensing site, considering the highly reversible protonization/hydrolysis equilibrium properties of anion species.

As the geometric optimized spatial conformations of coordinated structures shown in Figure 2b, anions tended to combine with tri‐pyridine group through the connection of Zn^2+^. The ionized Zinc atom was surrounded by nitrogen atoms and oxygen atoms, while the distance between nitrogen atom of tri‐pyridine and oxygen atom of acetate, was as close as 3.581 Å, which allowed the occurrence of charge transfer. Due to the close coordination bond distance, the hanging anions can participate in delocalized molecule orbital and related interactions.

According to further first‐principle calculation results in Figure 2c, for neutral state TBH, the transition density mainly distributed at the connecting Schiff‐base group while the strong adsorption sites mainly distributed around the tri‐pyridine group.

The mismatch of transition density distribution and adsorption site probably leads to a great waste of detection performance. By contrast, for TBH coordinated with acetic anion, both transition density and adsorption site distributed around the acetic anions, so that the guest molecules can be easily captured and lead to great influence on exciton transition process of emissive host structure, thus lead to better detection performance.

The tight coordination in theoretic models was well proved by XPS spectra. 406.5 eV N‐1s peaks of TBH‐Ac_2_ film demonstrated an obvious N─O bonding due to close distance, which cannot be observed in TBH film. After interacted with DCP vapor, such unique N‐1s peaks related with N─O interaction reversibly disappeared, indicated the acquisition of reversible sensing property.

Therefore, based on both theoretical and experimental results of the coordinated models, a new sensing mechanism with coordination hanging anions has been screened out, different from the conventional irreversible fluorescence sensing systems.

**Figure 3:**
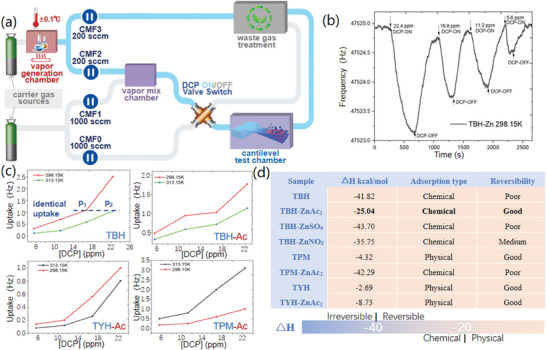
a) Diagram of adsorption analysis system with accurate gas concentration configuration. b) MEMS cantilever adsorption curve of TBH‐Ac_2_ for DCP vapor at 298.15K. c) Adsorption isothermal curves of tri‐pyridine derivative films. d) Adsorption Enthalpy and reversibility results of tri‐pyridine derivative films.

In order to effectively screen the designed coordination systems, reversible sensing process was systematically characterized with thermodynamic and kinetic adsorption analysis system in **Figure** **3**a.

A 5 ml chromatographic bottle containing DCP liquid, was settled in an accurate temperature‐controlled chamber for DCP vapor generation. The actual DCP vapor volatile speed was measured as 17.25 ± 1.41µg min^−1^ in 200 sccm carrier gas, by monitoring mass loss of the sample bottle, thus gained 22.4 ppm DCP vapor for further tests. The DCP vapor was accurately diluted with carrier gas in vapor mix chamber, under the modulation of four Coriolis mass flow meters, and a four‐way valve controlled the ON/OFF state of DCP vapor source for cantilever test chamber.

The resonance frequency changes of micro‐cantilever indicated the adsorption mass changes of the tri‐pyridine derivative films loaded on cantilever. (1.5 pg for 1 Hz) According to Figure 3b, the DCP vapor was reversibly adsorbed and desorbed on TBH‐Ac_2_ film, while the uptake value gradually decreased along with the decreasing DCP concentration at 298.15 K, from 1.77 Hz for 22.4 ppm, 1.03 Hz for 16.8 ppm, 0.95 Hz for 11.2 ppm, to 0.49 Hz for 5.6 ppm. Similarly, the uptake values of all tri‐pyridine derivative films were measured at two temperatures, for the adsorption isotherms in Figures 3c and S4 (Supporting Information). Consequently, DCP concentration values, demanded for identical uptake at different temperature, were obtained in Sheet S2 (Supporting Information), based on which the Enthalpy changes of adsorption process was calculated in Figure 3d. The sensing performance can be efficiently predicted before actual detection according to the results. Negative Enthalpy values of all films indicated the spontaneous adsorption process. In addition, Enthalpy value over −10 kJ mol^−1^ represented physical adsorption with weak adsorption, while Enthalpy value below −40 kJ mol^−1^ indicated violent chemical adsorption with poor adsorption reversibility.

For example, the −25.04 kJ mol^−1^ adsorption Enthalpy value of TBH‐Ac_2_ film located at typical reversible chemisorption range, while the of indicated poor adsorption reversibility. Due to the moderate adsorption Enthalpy values of TBH‐Ac_2_ film (−25.04 kcal mol^−1^) and TBH‐NO_3_ film (−35.75 kcal mol^−1^), they were predicted to be suitable for reversible DCP detection, while the −41.82 kJ mol^−1^ Enthalpy value TBH film is probably the reason of its irreversible DCP detection performance.

**Figure 4:**
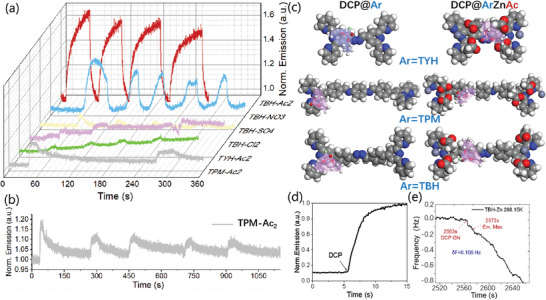
a,b) Fluorescent emission time‐curves of tri‐pyridine derivative films with multiple response‐recovery loops. c) The stable DCP adsorption state of TPM, TYH, TBH, and TPM‐Ac_2_, TYH‐Ac_2_, TBH‐Ac_2_ films after 10 ps dynamics process. d) Emission signal and e)adsorption signal changes of TBH‐Ac_2_ film after interacting with 22.4 ppm DCP vapor.

In application for fluorescence mode signal changes, the DCP vapor sensing curves of variable coordinated tri‐pyridine derivative films, shown in **Figure** **4**a,b, well agree with the analyzed models.

The TBH‐Ac_2_ film and TBH‐NO_3_ film both show good reversibility for DCP vapor detection, while TBH‐Ac_2_ film shows best emission enhancement, ≈2 times better than TBH‐NO_3_ film. In contrast, the TBH‐SO_4_ film, TBH‐Cl_2_ film, and TPM‐Ac_2_ film show poor reversible emission curves, agree with their over‐high adsorption Enthalpy values. The curve of TBH‐Cl_2_ film was even too poor to extract adsorption thermodynamic parameters. For TYH‐Ac2 film, the reversible curve with low adsorption capacity was also fit with its physical adsorption type with just 8.73 kcal mol^−1^ Enthalpy value. Thus, the tailored designing of highly reversible DCP fluorescence sensor TBH‐Ac_2_ has been successfully verified.

To further validate the intrinsic mechanism of the performance trends, the dynamics process of adsorption behavior has been analyzed. Figure 4c demonstrated the stable spatial structure of neutral TPM, TYH, and TBH and their acetic anion coordinated structures after thoroughly combined with DCP vapor. The positions of DCP vapor clearly indicated that the hanging acetic anion acted as an efficient active site in host‐guest interaction, instead of the tri‐pyridine group in the neutral models.

In addition, adsorption site of TYH‐Ac_2_ consists of four acetic anions, lead to excessive combination strength for DCP desorption. In comparison, TPM and TBH models own less hindered sites and performed larger adsorption capacity and better reversibility.

The efficient detection performance of TBH‐Ac_2_ film was also proved by time‐synchronized kinetic analysis, through comparing emission curve in Figure 4e and adsorption curves in Figure 4f. The fluorescence intensity took ≈9 s to reach its maximum after switching on the 22.4 ppm DCP vapor. Meanwhile only 0.106 Hz signal changes occurred in the adsorption curve in 9 s, far from the completion of whole adsorption process.

According to the micro‐cantilever frequency change after loading TBH‐Ac_2_ film, 613.28 Hz, the total amount of TBH‐Ac_2_ was calculated as 408.85pg/0.4 pmol, (M TBH‐Ac_2_ = 1034.17 g mol^−1^). 70.67 fg(0.4 fmol) DCP was adsorbed when TBH‐Ac_2_ film gained complete fluorescence mode signal change. Therefore, the emission signal of probe material can be sensitively triggered by 1/1000 equivalent quantity DCP molecule. The strong signal conversion efficiency is far more efficient than mass change itself, directly demonstrating the ultra‐high sensitivity of the hanging anion site as well as the fluorescence sensor technology.

Based on the above sensitive materials, efficient sensing performance for DCP was successfully achieved in both liquid phase and vapor phase.

**Figure 5:**
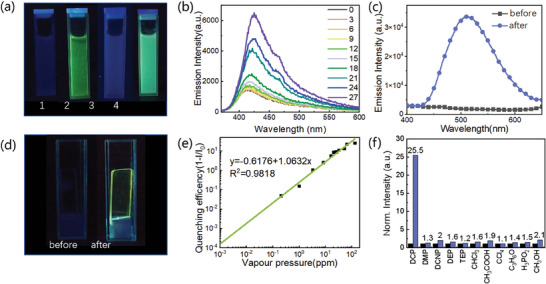
a) Fluorescence images of 1)TBH, 2)TBH with DCP, 3)TBH‐Ac_2_, and 4)TBH‐Ac_2_ with DCP. (3mL 10^−5^mol L^−1^ THF solution, 0.02 ml DCP addition) b) Emission spectra of TBH‐Ac_2_ solution with increasing DCP concentration. c) Emission spectra of TBH‐Ac_2_ films before and after DCP exposing. d) Fluorescence images of TBH‐Ac_2_ films before and after DCP exposing. e) Linear fitted enhancement rates of TBH‐Ac_2_ film. f) Enhancement rates of TBH‐Ac_2_ film in DCP and interferents.TBH‐Ac_2_ film performed a specific recognition for DCP with excellent selectivity. Among common interferents, methanol, acetic acid, acetone, chloroform, tetrachlorothane, and possible agents diethyl methyl phosphate (DMP), triethyl phosphate (TEP), diethyl cyanophosphate (DCNP), diethyl phosphate, and hypophosphoric acid.

As shown in **Figure** **5**a, after adding DCP, the fluorescence emission of TBH‐Ac_2_ solution rapidly converted from near‐dark to bright yellow‐green, more significant than the change in TBH solution. The emission of TBH solution, centered at 541 nm, gradually increased along with DCP addition, just doubled the initial intensity. In contrast, the emission of TBH‐Ac_2_ solution, centered at 425 nm, increased by ≈5 times in Figure 5b, which was far better than the TBH solution in Figure S5 (Supporting Information). The emission of TBH‐Ac_2_ solution gradually increased along with the increasing DCP concentration. The experimental detection limit of TBH‐Ac_2_ solution for DCP reached 58 nm, still with 4.5% enhancement rate, while the LOD was as low as 26.6 nm (1% fluorescence enhancement).

For on‐site application, TBH‐Ac_2_ film also shows comprehensive super performance for DCP vapor detection, from sensitivity, and selectivity to reversibility. In Figure 5c, TBH‐Ac_2_ film shows extremely weak fluorescence emission centered at 465 nm, and significantly enhanced after contacting with DCP vapor, with great wavelength redshift to 511 nm. The occurrence of bright yellow‐green emission was so significant that it can be directly observed by the naked eye, as shown in Figure 5d.

The emission intensity of TBH‐Ac_2_ film enhanced by 25.5 times in saturated DCP vapor, and just slighted enhanced in common interferents, including methanol (2.1 times), acetic acid (1.5 times), acetone (1.4 times), chloroform (1.6 times), carbon tetrachloride, and even structural‐similar phosphate compounds, phosphor diethyl methyl phosphate (DMP, 1.3 times), triethyl phosphate (TEP, 1.9 times), diethyl cyanophosphate (DCNP, 2 times), diethyl phosphate, and hypophosphoric acid (1.5 times).

According to Figure 5e, fluorescence enhancement rates of TBH‐Ac_2_ film exhibited good linearity for wide range DCP vapor concentration change with 0.98 R‐square value. The vapor phase detection limit reached 5.7 ppb, which is lower than the 7 ppb harmful threshold for human body. In addition, the film also shows rapid response and recovery speed for DCP vapor detection. The emission intensity rapidly reached 90% of maximum intensity in 10 s after contacting with DCP vapor, and also rapidly recovered to initial intensity in 20 s.

## 3 Conclusion

In conclusion, we developed a tailored mechanism screen and design strategy for super‐performed chemo‐sensors, assisted by structure‐effect analysis from theoretical simulation to MEMS analysis. Through three‐step tailored screen and design: Structure modeling; MEMS analysis and Performance verification, a novel mechanism was screened out for solving the challenging reversible nerve agent identification.

Hanging anions in a delocalized coordination structure, acted as reversible active site with efficient binding‐dissociation, instead of conventional irreversible pyridine groups. Detection performance of different coordination derivatives was predicted with first‐principle calculation results and MEMS adsorption parameters, and well supported by experimental fluorescence performance. TBH‐Ac_2_ was screened out with acetate hanging site with excellent reversibility, show reversible emission enhancement by 25.8 times with DCP, ultrasensitive detection limit, 26.6 nm in the liquid phase and 5.7 ppb in vapor phase, together with rapid response in 10 s, and recovery time in 20 s. Such tailored mechanism design strategy within structure‐activity‐associated screening will probably play an important role in developing high‐performance sensing system in the future.

## Conflict of Interest

The authors declare no conflict of interest.

## Supporting information



Supporting Information

## Data Availability

The data that support the findings of this study are available from the corresponding author upon reasonable request.
