# Supplementary material for: Tailoring Super‐Performed Chemo‐Sensor via Simulation‐Modeling and MEMS‐Screening
Source: Adv Sci (Weinh). 2025 Jan 7;12(8):2412937. doi: 10.1002/advs.202412937 (PMC11848570; doi:10.1002/advs.202412937)
Supplement: Supplementary file 1 — Supporting Information [file ADVS-12-2412937-s001.docx]

**Tailoring High-performed Chemo-Sensor via Simulation-modeling and MEMS-Screening**

Wei Xu^*†‡#^, Wukun Zhang^†#^, Zhengqi Shen^†^, Wenxing Xu^†^, Jianhao Zhao^†^, Huizi Li^†^, Qingguo He^†‡^, Yanyan Fu^*^^†‡^ and Jiangong Cheng^*†‡^

† State Key Lab of Transducer Technology, Shanghai Institute of Microsystem and Information Technology, Chinese Academy of Sciences, Changning Road 865, Shanghai 200050, China.

‡ Center of Materials Science and Optoelectronics Engineering, University of the Chinese Academy of Sciences, Yuquan Road 19, Beijing, 100039, China.

^#^ These authors are listed as co-first authors.

E-mail: windxu@mail.sim.ac.cn; fuyy@mail.sim.ac.cn; jgcheng@mail.sim.ac.cn;

Fax:+86-21-62511070-8934; Tel:+86-21-62511070-8933.

**Content**

1. Detailed calculation parameters and results 2

2. Adsorption kinetics data acquisition steps 3

3. Concentration calibration of DCP vapor 4

4. Thermodynamic parameters analysis results 5

5. Emission spectra and sensing results 7

6. Materials and methods 8

# 1. Detailed calculation parameters and results

Sheet S1 Parameter settings for geometry optimization and kinetic simulation

| Step 1.Forcite Geometry Optimization | Step 2.Forcite Dynamics |
| --- | --- |
| Ensemble: NVE  Quality: Ultra-fine  Forcefield: COMPASS II  Charges: Forcefield assigned  Quality: Ultra-fine  Summation method: Electrostatic: atom based; Van der waals: atom based | **Ensemble: NVE**  **Total simulation time: 50 ps**  **Quality: Ultra-fine**  **Forcefield: COMPASS II**  **Charges: Forcefield assigned**  **Quality: Ultra-fine**  **Summation method: Electrostatic: atom based; Van der waals: atom based** |

**ORCA input parameter for first-principle simulation：**

!M062X def2-TZVP def2/J def2-TZVP/C RIJCOSX tightSCF miniprint

%maxcore 3000

%pal nprocs 56 end

%tddft

nroots 10

printlevel 3

end

* xyz 0 1

Figure S1. HOMO and LUMO orbitals of TBH and TBH-Ac2 models.


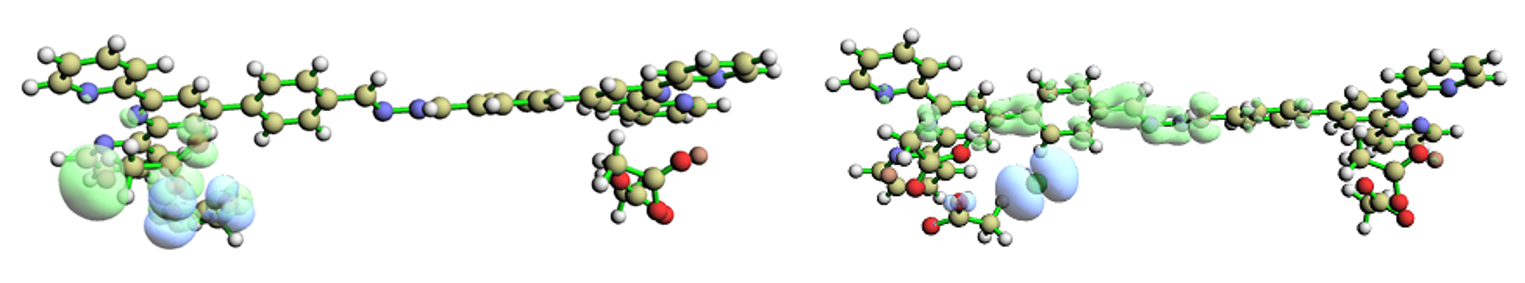


Figure S2. S1 state hole-electron distribution of TBH-Ac2

# Adsorption kinetics data acquisition steps

1. The solution of the prepared sensing material is coated on the micro-cantilever with a manipulator under a microscope, and the solvent is completely volatilized after drying and aging at 40℃ for 8 hours.
2. Pure DCP was added to a 5 ml Chromatographic bottle, sealed by a polydimethylsiloxane (PDMS) film.
3. Weigh the Chromatographic bottle, then place it in thermostatic and inert gas flow atmosphere, then take it out and weigh it at regular intervals. Plot the Mass curve of the sample bottle with increasing heating time and then the actual DCP concentration in carrier gas can be calculated.

(4) Test resonance frequency and Q value (indicating signal quality) of empty cantilever and cantilever loaded with sensitive materials.

(5) Connect the gas circuit, control the DCP vapor concentration, and get the sensor frequency change map at different temperatures;

$$\Delta H^{\circ}=\frac{{RT}_{1}T_{2}}{T_{2}-T_{1}}(\ln\frac{p_{1}}{p_{2}})$$

1. The enthalpy change was calculated by the equation above.Linear fit the p-p/V curve equation: p/V=Ap+B,here 1/V∞=A(cm^-3^);1/KV∞=B.

The standard equilibrium constants such as Gibbs free energy variation and entropy variation are calculated by equations:

K=A/B，K^o^=K*p^o^, ∆G°=-RTlnK^o^, ΔG°=ΔH-TΔS°

# 3. Concentration calibration of DCP vapor


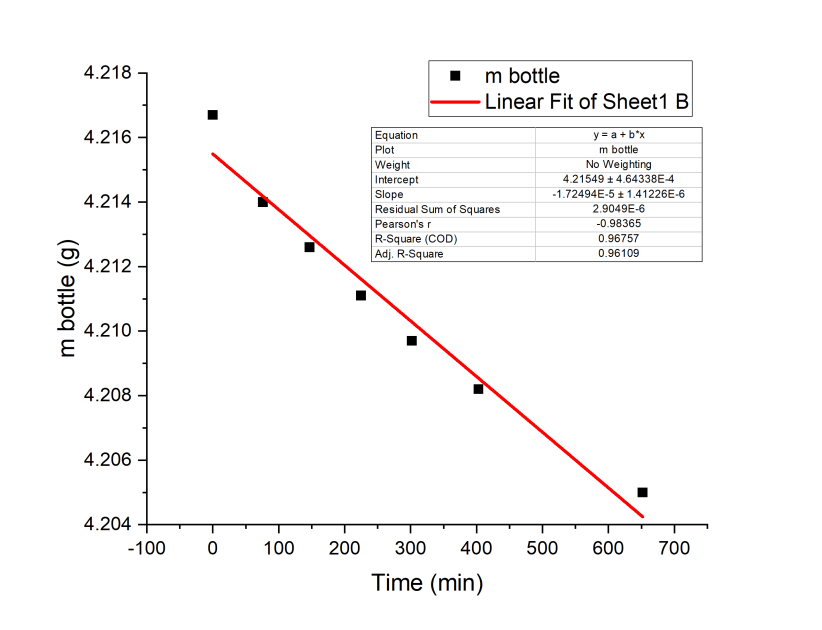


Figure S3. Mass curve of the sample bottle with increasing heating time

Long time tracking at 200 sccm gas flow (i. e., 200 ml/min)，the bottle mass decreased steadily with time，the slope of linear fitted line was 1.72494*10^-5^ ± 1.41226*10^-6^ g/min,hence the loss rate was 17.25±1.41μg/min;

The volatilization amount of DCP per minute was 17.25μg，The relative molecular mass of the DCP is 172.5 g/mol，so that n(DCP)= 0.1μmol，V(DCP)= 2.24μL，[DCP] =22.4 ppm

Adjust the concentration at different set proportions from N_2_：DCP=0：100, N_2_：DCP=25：75, N_2_：DCP=50：50 to N_2_：DCP=75：25, and obtain the spectrum of cantilever frequencies at different temperatures and different gas concentrations.

The frequency of loaded micro-cantilever was just the value measured with N_2_：DCP=100：0, with zero DCP vapor concentration. With detailed calculation, the partial pressures of DCP concentration at 22.4 ppm, 16.8 ppm, 11.2 ppm and 5.6 ppm are respectively 22.4 Pa, 16.8 Pa, 11.2 Pa and 5.6 Pa。


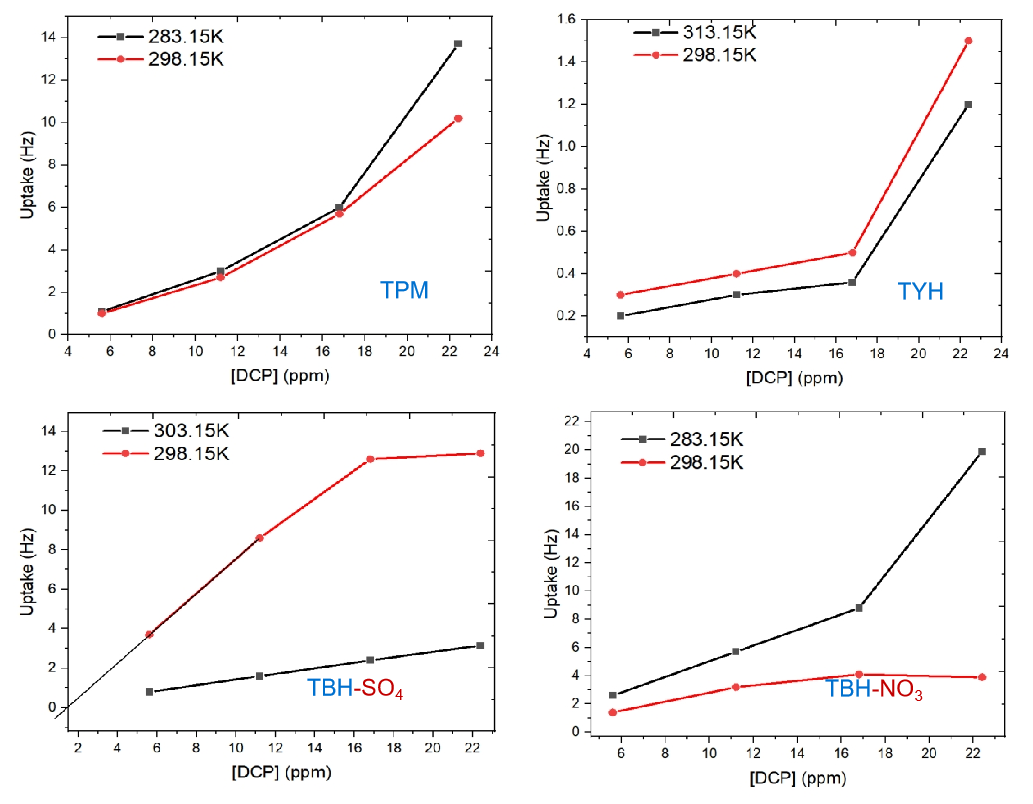


Figure S4. Adsorption isothermal curves of additional tri-pyridine derivative films.

# 4. Thermodynamic parameters analysis results

Sheet S2. Frequency and uptake values measured by MEMS micro-cantilever.

| **Sample** | **Initial Freq./Hz** | **Load Freq. /Hz** | **Temp/K** | **Freq. changes in DCP carrier gas/Hz** | | | |
| --- | --- | --- | --- | --- | --- | --- | --- |
| **DCP/ppm** | **/** | **/** | **/** | **22.4** | **16.8** | **11.2** | **5.6** |
| **TBH** | **48957.91** | **48836** | **298.15** | **2.51** | **1.1** | **0.7** | **0.32** |
|  |  |  | **313.15** | **1.06** | **0.59** | **0.23** | **0.12** |
| **TBH-Ac_2_** | **47539.14** | **47434.2** | **298.15** | **1.77** | **1.03** | **0.95** | **0.49** |
|  |  |  | **313.15** | **1.14** | **0.72** | **0.59** | **0.33** |
| **TBH-SO_4_** | **47325.83** | **46196** | **298.15** | **12.9** | **12.6** | **8.6** | **3.7** |
|  |  |  | **313.15** | **3.15** | **2.4** | **1.6** | **0.8** |
| **TBH-NO_3_** | **48936.43** | **48338.23** | **283.15** | **19.9** | **8.8** | **5.7** | **2.6** |
|  |  |  | **298.15** | **3.9** | **4.1** | **3.2** | **1.4** |
| **TPM** | **48745.91** | **48349.46** | **283.15** | **13.7** | **6** | **3** | **1.1** |
|  |  |  | **298.15** | **10.2** | **5.7** | **2.7** | **1** |
| **TPM-Ac_2_** | **48731.33** | **48672.59** | **298.15** | **1** | **0.6** | **0.25** | **0.18** |
|  |  |  | **313.15** | **3.1** | **2** | **0.8** | **0.5** |
| **TYH** | **46616.29** | **46320.15** | **298.15** | **1.5** | **0.5** | **0.4** | **0.3** |
|  |  |  | **313.15** | **1.2** | **0.36** | **0.3** | **0.2** |
| **TYH-Ac_2_** | **47462.14** | **46308.80** | **298.15** | **1** | **0.56** | **0.2** | **0.14** |
|  |  |  | **313.15** | **0.8** | **0.26** | **0.12** | **0.08** |

For the samples with too weak adsorption signal at 313.15K, 283.15K adsorption signal was measured instead.

Sheet S3. Enthalpy calculations of TBH-Ac2 and TBH films

| sample | TBH-Zn | | TBH | |
| --- | --- | --- | --- | --- |
| T/K | 298.15 | 313.15 | 298.15 | 313.15 |
| P/pa | 5.6 | 9.1 | 5.6 | 12.6 |
| ΔH/kcal/mol | -25.04 | | -41.82 | |

Sheet S4. Entropy change calculation table of TBH-Ac2 and TBH film

| Plot | S2-25℃ | S2-40℃ | S1-25℃ | S1-40℃ |
| --- | --- | --- | --- | --- |
| Intercept | 12 | 12.1 | 12.6 | 13.2 |
| Slope | 1.15 | 1.16 | 0.603 | 0.425 |
| K | 0.096327 | 0.095535 | 0.047827 | 0.032263 |
| K_o_=K*p^o^ | 9760.35 | 9680.09 | 4846.13 | 3269.09 |
| ∆G°=-RTlnK^o^ | -21.63 | -21.61 | -20.96 | -20.40 |

The negative adsorption Gibbs free energy values, gained in Sheet S4, also indicated the spontaneous adsorption process in atmosphere conditions.

Sheet S5. Calculation process of adsorption enthalpy values of triplet pyridine coordination fluorescent materials

| Sample | T/K | p/Pa | △H kcal/mol | Adsorption type | Reversibility |
| --- | --- | --- | --- | --- | --- |
| TBH | 297.15 | 5.6 | -41.82 | Chemical | Poor |
|  | 313.15 | 12.6 |  |  |  |
| TBH-Ac_2_ | 297.15 | 5.6 | -25.04 | Chemical | Good |
|  | 313.15 | 9.1 |  |  |  |
| TBH-SO_4_ | 297.15 | 2.4 | -43.70 | Chemical | Poor |
|  | 313.15 | 5.6 |  |  |  |
| TBH-NO_3_ | 297.15 | 8.4 | -35.75 | Chemical | Medium |
|  | 313.15 | 16.8 |  |  |  |
| TPM | 297.15 | 10.3 | -4.32 | Physical | Good |
|  | 313.15 | 11.2 |  |  |  |
| TPM-Ac_2_ | 297.15 | 7.4 | -42.29 | Chemical | Poor |
|  | 313.15 | 16.8 |  |  |  |
| TYH | 297.15 | 16.8 | -2.69 | Physical | Good |
|  | 313.15 | 17.7 |  |  |  |
| TYH-Ac_2_ | 297.15 | 16.8 | -8.73 | Physical | Good |
|  | 313.15 | 19.9 |  |  |  |

# Emission spectra and sensing results

Figure S5. Emission spectra of TBH solution with increasing DCP vapor concentration.

Figure S6. UV-vis absorption spectra of TBH-Ac_2_ film.

Figure S7. Changes in the UV-vis absorption spectra with Zn^2+^ addition into TBH solution.

# Materials and methods

**Experiment reagents and equipment:**

All reagents and solvents are purchased through commercial sources and used as received. KW-4A desktop homogenizer (Institute of Microelectronics, Chinese Academy of Sciences) was used for sensing film preparation.

The NMR and mass spectrum tests were conducted with Bruker DRX 500 superconducting NMR instrument, Thermo Fisher Scientific Orbititrap-FTMS and LTQ FT Ultra mass spectrometer at Shanghai Institute of Organic Chemistryrespectively. The spectral test was conducted with HORIBA Fluoromax 4 Plus fluorescence spectrometer and JASCO V-670 UV-visible absorption spectrometer. Theoretical modeling and calculation are conducted with Materials studio 8 and ORCA 5.0.2. Adsorption cantilever data were recorded using a Cantilever Thermogravimetric Analyzer (model：Loc-TGA 3000), produced by Xiamen High-End MEMS Technology Co., Ltd.

Figure S8. Molecule structure of TBH coordinated system.

**Synthesis of TBH：**1.00 g 4-([2,2':6',2''-terpyridin]-4'-yl) benzaldehyde was dissolved in 300 ml ethanol solvent, and added into 500 mL double-neck bottle together with 100 μL hydralazine alhydrate. After 5h reaction at 80℃ with condensation reflux, heat-filter, washing in ethanol solvent, and vacuum drying, 660.9 mg TBH was gained as light yellow solid powder with 66.1% yield.

HRMS-AP-MALDI (m/z): [M+H]^+^ calculated for [C44H31N8]^+^, 671.26, found 671.2673.

^1^H-NMR (600 MHz, THF) δ8.64-8.62 (m, 6H), 8.62-8.60 (m, 4H), 8.06 (d, J = 8.2 Hz, 4H), 7.98 (d, J = 8.2 Hz, 4H), 7.82 (td, J = 7.7, 1.7 Hz, 4H), 7.31-7.27 (m, 8H).

**Synthesis of TBH-Zn：**6.70mg TBH was dissolved in 5 mL THF solvent, 4.40mg zinc acetate was dissolved in 5 mL methanol. Then TBH THF solution was slowly added into zinc acetate methanol solution to form TBH-Zn coordination complex solution, in which TBH:Zn^2+^=1:2.

**Preparation of Sensitive solution and film**

Surface optimized 10×20 mm quartz plates were gained with 10 minutes UV ozone treatment. 11.10mg/L. TBH solution was dropped on quartz plate, handled with homogenization machine with two step procedure: 200 rpm for 10 seconds, and 2000 rpm for 20 seconds. The gained sensitive films were further vacuum dried for 1 hour at 40℃.

**Stability of sensing film**

The TBH-Ac_2_ film show the good film stability after long term storage.

After storage with over 3 months, the sensing film remained reversible sensing performance for saturated DCP vapor with 25.2 times signal enhancement and good reversibility similar to new-fabricated films.


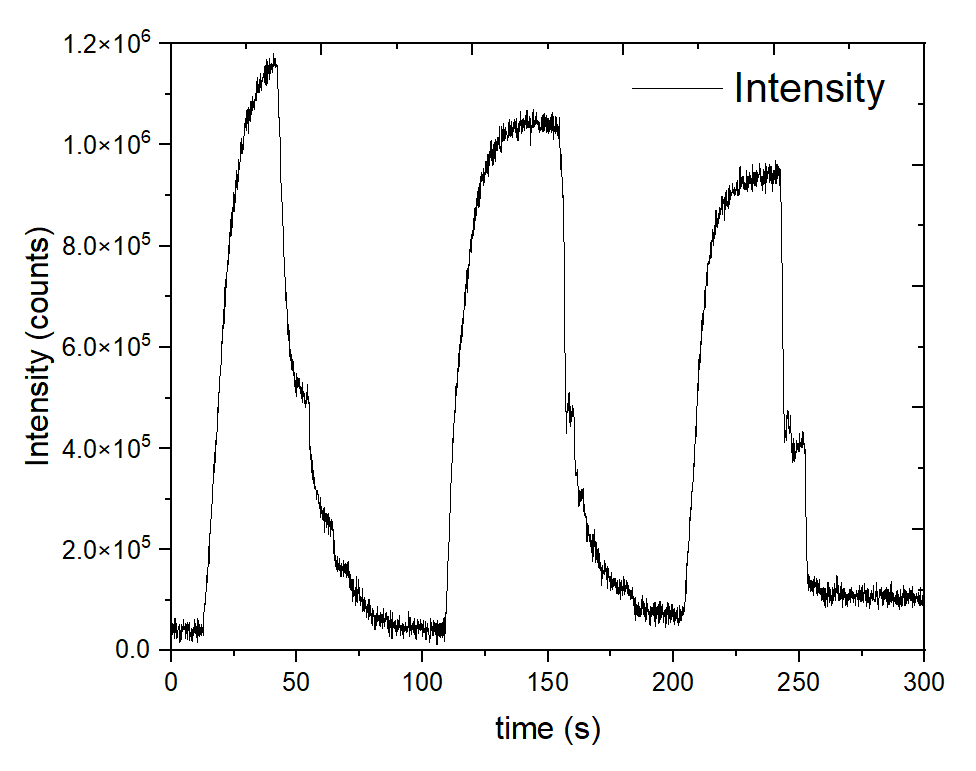


Figure S9. Reversible detection process of TBH-Ac_2_ film after 3-month storage after fabrication.


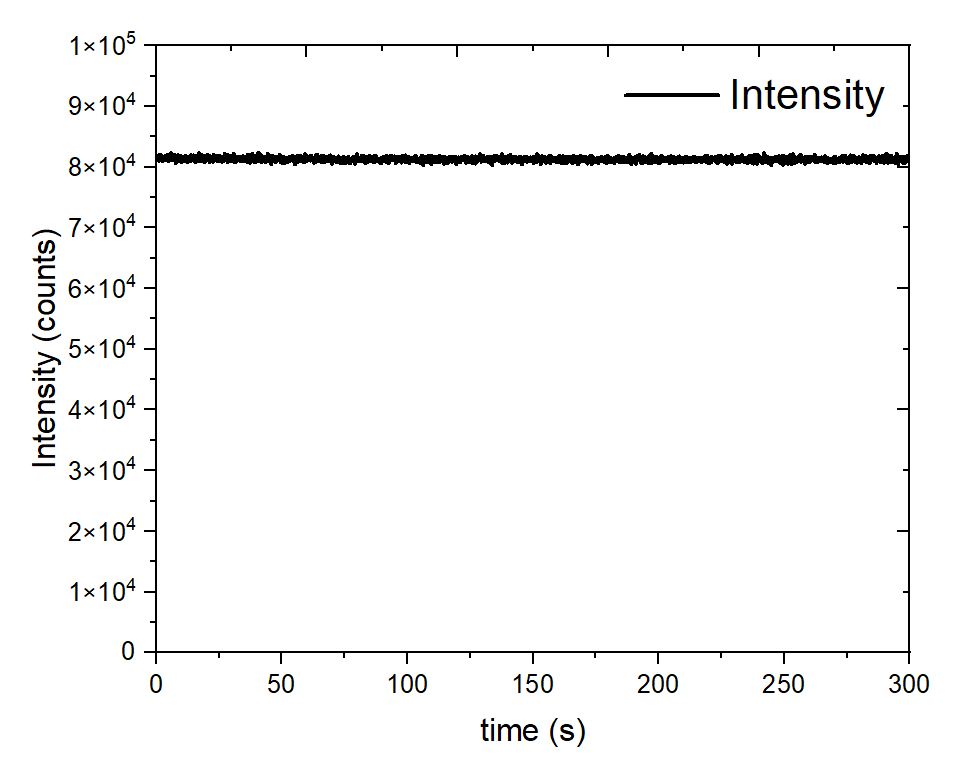


Figure S10. Emission intensity curve of TBH-Ac_2_ film in 300s under 29.4nm*29.4nm slit width excitation

The emission intensity loss is below 2‰ in 300s under extremely strong excitation light with largest slit width at 29.4nm*29.4nm with FL-max 4p Fluorescence spectrometer.

**Sheet S6.** Comparison of the advantages and disadvantages of different DCP detection.

| Fluorescent Probe | LOD | Sensing Mechanism | Reversibility | Ref. |
| --- | --- | --- | --- | --- |
| HDB | 6.08 ppb | Twisted Intramolecular Charge Transfer (TICT) | × | [1] |
| ESIPT Reporter | 1.2 ppm | Excited State Intramolecular Proton Transfer (ESIPT) | × | [2] |
| Cu^2+^@UiO-66-NH_2_/OPD | 6.65 ppb | Ligand Metal Charge Transfer (LMCT) | × | [3] |
| CdAgAlloy@Pdots | 0.85 nM | Redox Interactions | × | [4] |
| TBH-Ac_2_ | 5.7 ppb | Coordination hanging site (supramolecular Charge Transfer ) | √ | This work |

1. Adv Fiber Mater, 5(2023) 1632-42,
2. Small Methods，8(2024) 2301048,
3. Sensors and Actuators: B. Chemical 396 (2023) 134553.
4. J Mater Chem C, 9(2021) 16721-31,
